# A Case Report on Endovascular Aortic Repair Rupture

**DOI:** 10.7759/cureus.9209

**Published:** 2020-07-15

**Authors:** Jose Rubero, Thor S Stead, Latha Ganti

**Affiliations:** 1 Emergency Medicine, University of Central Florida College of Medicine, Orlando, USA; 2 Emergency Medicine, Alpert Medical School of Brown University, Providence, USA; 3 Emergency Medicine, Envision Physician Services, Nashville, USA; 4 Emergency Medicine, University of Central Florida College of Medicine/Hospital Corporation of America Graduate Medical Education Consortium of Greater Orlando, Orlando, USA; 5 Emergency Medical Services, Polk County Fire Rescue, Bartow, USA

**Keywords:** endovascular aneurysm repair, aaa

## Abstract

Endovascular repair of an abdominal aortic aneurysm (AAA) is a widely accepted alternative to open surgical AAA repair. A ruptured AAA is among the emergency surgeries with the highest risk of death, with an overall mortality rate close to90%. However, the classic symptom triad for ruptured AAAs of hypotension, a pulsatile mass, and abdominal/back pain is seen in only in 25% to 50% of affected patients. Thus, many present with symptoms and signs that suggest adifferent diagnosis. Recognizing uncommon presentations and limitations of imaging and interpretation, in addition to clinical gestalt, can save many lives. This report discusses an unusual case involving a previously repaired AAA presenting with acute rupture at the endograft site.

## Introduction

An abdominal aortic aneurysm (AAA) is defined as a dilation of the subdiaphragmatic aorta to a diameter greater than 3.0 cm. AAAs are found in up to 7% of men and 1% of women aged 50 years and older, particularly smokers [1]. Up to 80% of aneurysmal ruptures can occur in previously undiagnosed aneurysms [2]. Most undiagnosed patients with AAA remain asymptomatic unless they develop a complication. Rupture, the most common complication of AAA, is also the most lethal [3]. Most AAAs will rupture into the retroperitoneal cavity, rendering ultrasound for a quick diagnosis ineffective. Depending on the rupture site, the presentation varies and may be very nonspecific, one of the reasons why ruptured AAA is frequently misdiagnosed [4].

Currently, endovascular aortic repair (EVAR) is the main method used for AAA repair with lower rates of morbidity, mortality, and a shorter hospital stay compared to open repair [5,6]. Patients undergoing EVAR encounter unique complications, such as endoleaks, rupture, and infection. For these reasons, postprocedure surveillance CT scans are recommended at approximately one month, six months, twelve months, and annually thereafter [7]. It is well understood that patients with poor follow-up have a higher rate of complications [8]. Re-rupture after endovascular repair is a rare event that usually will be secondary to multiple risk factors, including larger aneurysm size, poor sealing zones, female gender, presence of aorto-enteric fistula, and stent-graft infection [7]. Infection of an endograft is rare; however, it has a very high mortality when present.

## Case presentation

A 70-year-old male with medical history of atrial fibrillation, hypertension, coronary artery disease, tobacco abuse, and AAA with repair presented to our emergency department (ED) as a transfer from another facility for suspected ruptured AAA. According to the transferring doctor, the patient was at a follow-up visit for multiple lung nodules and a positron emission tomography (PET) scan. The patient was complaining of epigastric pain at that visit, and the preliminary results of the PET scan showed a possible ruptured AAA. His aneurysm was first noted six years prior with mild dilation, two years afterwards it measured 4.2 cm, and by the end of that year it measured 5.2 cm, at which time the patient went for elective repair. The patient stated he had been experiencing intermittent abdominal pain with back pain for about three weeks. Today, his symptoms were worse and located in the epigastrium with radiation to the back. Of note, the patient was on metoprolol and warfarin for atrial fibrillation, with a last known international normalized ratio (INR) of 3.7 the previous month. The patient denied vomiting, diarrhea, chest pain, cough, fever, diarrhea, melena, hematemesis, fever, leg swelling or numbness, dizziness, or shortness of breath.

His initial vital signs were within normal limits with the exception of tachycardia at 113 bpm. His physical exam showed diffuse abdominal pain with no pulsatile masses felt on palpitation, pale lower extremities with good and equal overall peripheral pulses, and no other abnormalities. His electrocardiogram showed atrial fibrillation with no acute ischemic changes. Laboratory results showed mild anemia with a hemoglobin of 11.1 g/dL, mild hyponatremia, INR of 4.3, and nonspecific leukocytosis.

**Figure 1:**
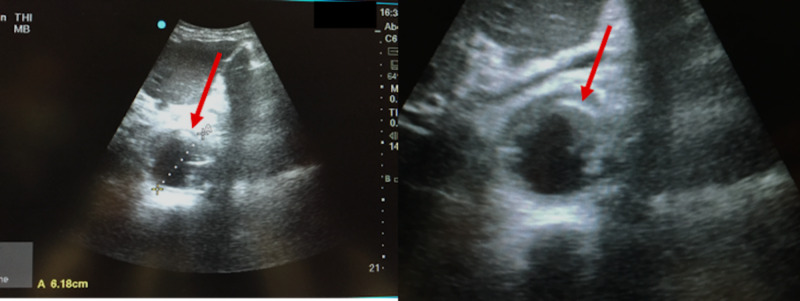
Bedside ultrasonography demonstrating aortic dilation of 6.2 cm with no free fluid in abdomen.

A bedside ultrasound showed aortic dilation of 6.2 cm with no free fluid in abdomen (Figure 1).

**Figure 2:**
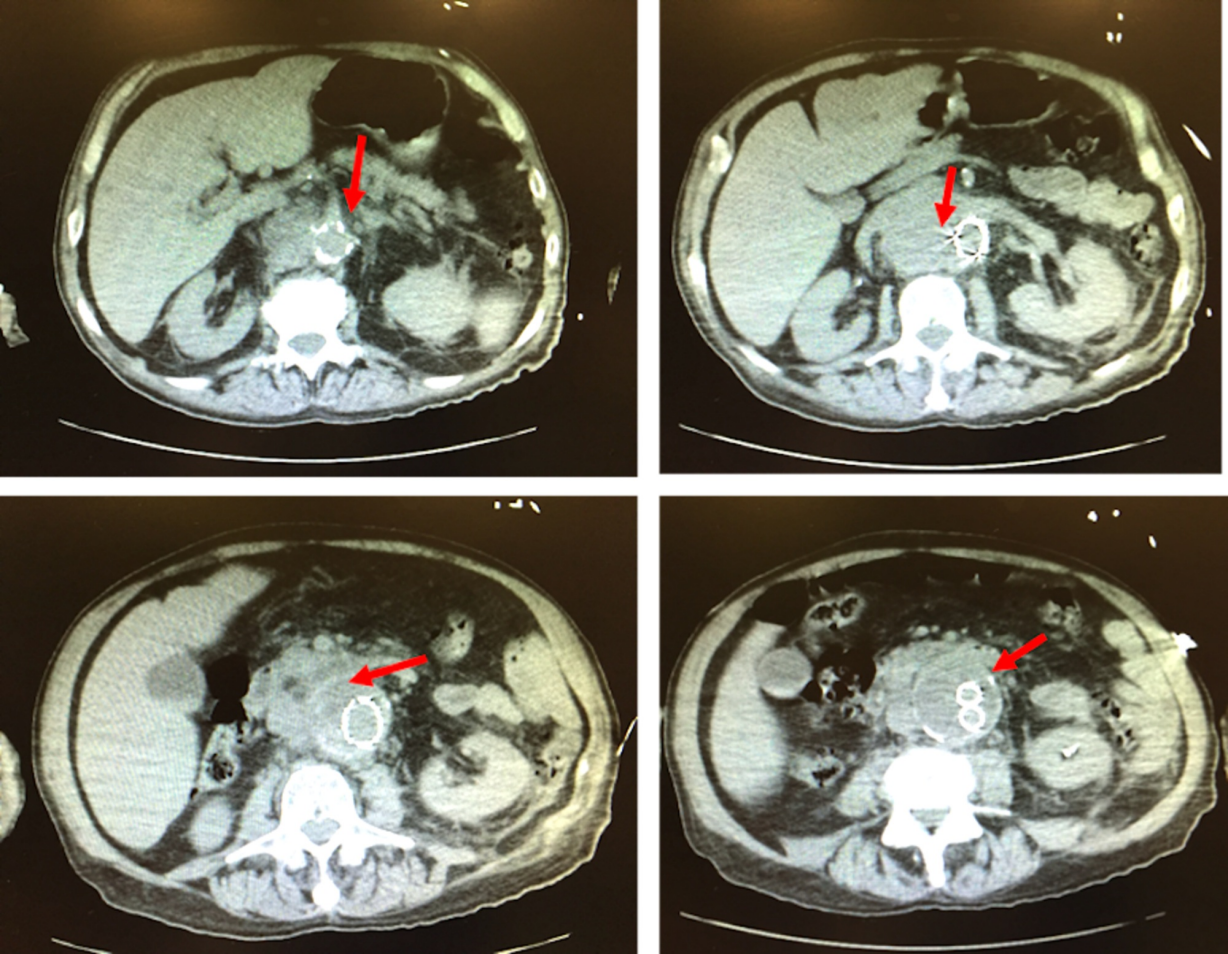
Noncontrast CT scan demonstrating false lumen of abdominal aortic aneurysm (red arrows).

The vascular surgeon was called to the bedside. The decision was made to perform a noncontrast CT (Figure 2).

The emergency physician and vascular surgeon were at the bedside in the CT suite. After reviewing the images and taking the patient’s presentation into consideration, the patient was taken to the operating room for acute rupture at endovascular graft site.

An interesting twist to this case followed. The radiologist contacted the emergency physician to report CT findings: mass effect to the right of the aorta at the level of the right kidney with anterior displacement or invasion of the inferior vena cava. According to the report, the density made hemorrhage less likely, and thus findings were concerning for a neoplastic process. The radiologist recommended a follow-up CT angiography and venography and advised not to send the patient to the operating room, as the CT imaging in his view made it unlikely that the patient had an acute rupture. An attempt was made to contact vascular surgeon, but he was already in the operating room with the patient on the surgical table. Fortunately, upon conclusion of surgery, the patient was indeed diagnosed with an acute rupture at the endovascular site due to a concurrent endograft infection.

## Discussion

Compared to traditional open repair of AAA, EVAR represents a far safer, minimally invasive technique that is sometimes performed as an outpatient procedure. EVAR helps to avoid the hemodynamic and acid/base fluctuations of open repair that exert a huge metabolic strain on the (typically older) patients [9]. Since the approval of endovascular stents for this purpose, the United States has seen rates of EVAR procedures increase sixfold [10]. In geriatric patients, EVAR has proven to avoid common complications such as delirium (following vascular surgery), and expands treatment options for those who would not be candidates for open repair [11].

Prognosis is very good in patients who have not yet suffered a rupture and are undergoing elective repair of AAA, with mortality at virtually zero. Among all patients (including those with rupture), EVAR perioperative mortality is on average 3.3 times less than open repair [12]. These findings suggest that EVAR is a preferred option to open repair of AAA. However, EVAR patients are more likely to require surgical re-intervention down the road than patients who underwent an open repair; therefore, the age and surgical status of the patient must be taken into consideration when considering treatment options. Due to the immediate benefits and much lower risk of mortality with each procedure, EVAR is typically preferred in older patients [12].

Another potential complication in patients with ruptured AAA is the increased incidence of acute kidney injury (AKI) stemming from fluid depletion, increase in circulating pyrogenic molecules, and induced oxidative stress [13]. This is marked by a postoperative 30% increase in serum creatinine. Retrospective studies have shown variable incidence rates of AKI following EVAR, from 3% to 23%, and the most contemporary study reports a 10% incidence of AKI [14-18]. Therefore, postoperative management and follow-up are of critical importance in cases of AAA. Patient labs should also be monitored following surgery and during outpatient follow-up to rule out AKI.

## Conclusions

The rupture of an aneurysm is a potentially life-threatening complication of a dilated abdominal aorta with relatively high mortality. When a prior repair has been performed the presentation may be even more challenging. A patient with adequate follow up may have less risk of having an unnoticed rupture or endoleak. In this case, we discuss a patient with recent AAA repair and no prior complications, who now presents with three weeks of unreported symptoms. This case demonstrates the difficulties in initial diagnosis and management that initially arise in this clinical presentation. In this example, we also see the need for surgical repair highlighted by clinical gestalt in opposition to radiological interpretation.
